# Supplementary material for: Mycotoxins of Concern in Children and Infant Cereal Food at European Level: Incidence and Bioaccessibility
Source: Toxins (Basel). 2022 Jul 15;14(7):488. doi: 10.3390/toxins14070488 (PMC9317499; doi:10.3390/toxins14070488)
Supplement: Supplementary file 1 [file toxins-14-00488-s001.zip › toxins-1760726-supplementary.pdf]

Mycotoxins of Concern in Children and Infant Cereal Food at European Level: Incidence and Bioaccessibility

Cheila Pereira, Sara C. Cunha, and José O. Fernandes

File S1: Methods for Mycotoxin Analysis in Food

Sampling

Sampling is especially difficult with mycotoxins due to their heterogenous distribution in contaminated raw and processed food products [1-3]. For this reason, there is a common sampling procedure established by EU for the official control of AFs, OTA, Fusarium toxins and PAT in baby food products and processed cereal-based foods, for infants and young children, Community Regulation No. 401/2006. In summary for raw cereals, processed cereals and cereals products marketed for children, 100 incremental samples should be taken in lots above 50 tones, resulting in an aggregate sample of 10 kg; for lots below 50 tones, 10 to 100 incremental samples should be taken, resulting in an aggregate sample of 1 to 10 kg, and for lots at retail stage, there should be a minimum aggregate sample of 1 kg. The primary sample must be ground and mixed to ensure that the analytical portion has the same concentration of mycotoxin as the original sample.

Sample preparation

Sample preparation techniques and detection methods for mycotoxins assessment in infant cereal matrices published in last years are summarized in Table S1. The homogenised sample, usually, goes through an initial step of sample pre-treatment, where the mycotoxins are extracted, the resulting extract is purified, concentrated (optional), and then occurs the separation and detection [4, 5]. The goal is to obtain an extract as clean as possible, free of any matrix components that may be co-extracted, so reducing matrix effects, containing the mycotoxins under study in the highest concentration possible. There are several extraction/clean-up techniques proposed and the choice must be in careful consideration the type of matrix, the physicochemical characteristics of the analytes, and the chosen method for separation and detection [3, 4]. Solid-phase extraction (SPE) is a method most used for clean-up and concentration in solid matrices after the extraction of mycotoxins with a mixture of organic solvents, such as acetonitrile (ACN), methanol, and water. By using the extract solvent as an eluting solvent, the sample is extracted, concentrated, and purified in one step with the SPE approach [4, 6]. It is a safe, efficient and reproductible technique, but with some limitations, because it is not possible to use a single cartridge for all mycotoxins, and the performance is altered by pH, solvent use, and ionic strength of the sample [7]. For the extraction of FB1 and FB2 mycotoxins from breakfast cereals for children, Assunção and co-workers [8] used SAX SPE cartridge, with a good linear response in the UPLC-MS analysis, as it is possible to see in Table S1. Gotthardt [9] team also used this clean-up procedure after the extraction of Alternaria toxins from cereal-based baby food, with a HyperClone BDS-C18 column for AOH, AME, TEN, ATX I, ATX II, STTX III, and ALTP and a Gemini-NX C18 column for TA.

Due to the necessity of using less solvents and having more selective techniques, other methods have been developed. The more specific are based on immunoaffinity materials

[10]. Immunoaffinity columns (IAC) are a specific mode of solid-phase extraction, based on antibody-antigen interactions [4, 6]. This technique is highly specific and rapid, resulting in purer extracts with minimal contamination of unwanted matrix components and allowing low limits of quantification (LOQ). However, it is an expensive process, the column only enabling one use for a specific compound or group of compounds, and requiring experts for its practice [11]. As showed in Table S1, Juan et al. [12] used IAC as a clean-up method in the extraction of OTA, AFB1 and AFM1, when analysing infant formulas and baby food. Assunção and co-workers [8, 13], and Martins and colleagues [14] also used this type of clean up method for determination of AFB1, AFB2, AFG1, AFG2, AFM1 and OTA in breakfast cereals, cereal-based children food. In 2015, Hampikyan and colleagues used high performance liquid chromatography coupled to a fluorescent detector (HPLC-FD) as a confirmation method of the results obtained by Enzyme linked immunosorbent assay (ELISA). After extraction of OTA with a mixture of ACN:water (60:40, v/v) from baby food samples, they used for clean-up OchraTest® IAC. More recently, Herrera [15], used AflaTest and DonTest, Vicam®, IAC in the extraction process of AFs and DON from baby food samples. In multifunctional columns (MFC) the purification process is only one step. There are various commercial MFCs available, and in general, it is a fast and easy method because column pre-conditioning or rising is not necessary. Also these columns eliminate the errors of irreversible adsorption or premature elution of the analytes [16]. The drawback with MFCs is the fact that columns are single use, therefore sample concentration is not possible, and the purification step is not always effective with more complex matrices [7]. Pereira et al. (2015) [17] compared three different clean-up procedures for the analysis of TCs in cereal-based baby food samples, d-SPE, Multistep and IAC. Compared with other procedures d-SPE was easier to perform, very quick, inexpensive, and used low amounts of organic solvents (Table S1) .

A more economic approach than those mentioned above is QuEChERS (Quick, Easy, Cheap Effective Rugged and Safe). It is based in the extraction of the analytes with ACN in the presence of inorganic salts (MgSO4 and NaCl) followed by a dispersive SPE clean-up, where different sorbents can be used depending on the matrix and analyte [18-20]. Pereira et al. [17], used a modified QuEChERS extraction with d-SPE clean-up, TriSilTBT derivatization and GC-MS detection for determination of TCs in cereal-based baby-food samples. The same method was used by Assunção et al. [8, 13], and Martins and team [14], as referred in Table S1.

Separation and Detection methods

ELISA is the most frequently method used for the screening of mycotoxins [2, 6]. There are commercial kits of ELISA for every regulated mycotoxin, which are easy to use and present a good linearity analytical range [1]. While it is a fast, simple, and easy to use technique, cross-reactivity may occur, and kits can only be used once. Positive ELISA results should be confirmed by a chromatographic method, for accurate quantitative results [6, 21, 22]. Hampikyab et al. [23] used competitive ELISA as a screening method in the analysis of OTA in cereal-based baby foods. The positive samples were confirmed with HPLC-FD and the difference of mean range between ELISA and HPLC methods was 0.07 (0.20%), therefore confirming that ELISA permitted a fast and easy routine screening test. Capei and co-workers [24] used a commercial kit, I'screen Ochra-cod.OR 360, for the determination of OTA in breakfast cereals and cereal-based sweet cakes (Table S1).

Liquid chromatography (LC) is the most relied upon method of separation in mycotoxin analysis, with both normal phase and reverse phase columns. However, because

reverse-phase columns are easier to use and water-based mobile phases are less toxic, most separations are performed with them. Usually, the preference falls on C18 columns with water and MeOH/ACN mixtures as mobile phase [1, 5, 25]. In Table S1, it is possible to see that the majority of the analysis used HPLC method for the detection and quantification of mycotoxins in cereal-based baby/infant/children food. Different detectors can be used, UV detector has been used for the detection of PAT [12], while AFs and OTA, due to their chemical properties are usually detected by a fluorescent detector (FD) [8, 12-15].

LC coupled to MS detector has been also commonly used for mycotoxin analysis, even though it is expensive and requires trained experienced users [1, 26]. The use of multiple MS analysers coupled to a detector (LC-MS/MS), that can also lead to a higher separation capacity, is done via atmospheric pressure ionization (API) techniques, such as electrospray ionization (ESI), atmospheric pressure chemical ionization (APCI), or atmospheric pressure photoionization (APPI) [27, 28]. As it is possible to see in Table S1, MS was used by Juan et al. [12] for the analysis of TCs, ENs and BEA, by Assunção and co-workers [8] for the detection and quantification of FB1 and FB2, by Assunção et al. [13, 14] in the analysis of FB1, FB2 and ZEA, by Gotthardt [9] in the detection of Alternaria toxins, by the team of Postupolski [29] in the analysis of TCs, FB1 and FB2, and by Braun et al. [30] for the detection and quantification of several regulated and emerging mycotoxins. Other tandem MS detectors can be used with LC, such as ion trap (IT), triple quadrupole (QqQ), and combined quadrupole linear ion trap (QTRAP) [10, 22, 31].

GC methods have been also applied in the analysis of multi-mycotoxins. However, their use implies the derivatization of analytes to ensure higher volatility and lower polarity of many compounds [25]. The analysis of TCs mycotoxins is performed with GC-MS detection coupled with a modified QuEChERS extraction in the studies of Pereira et al.[17], Assunção et al. [8, 13], and Martins and co-workers [14] (Table S1).

Table S1 - Methods for analysis of mycotoxins in cereal-based food for infants and children, in Europe (2014-2021).

Matrix	Mycotoxin	Sample pre-treatment		Method			Reference
		**Extraction**	**Clean-up**	**Derivatization**	**Detection method**	**LOD; LOQ (µg/kg)**	
Infant formulas and baby food	OTA	ACN:water 60:40 (v/v)	Ochratest IAC	-	HPLC-FD	0.050; 0.150	Juan *et al*., 2014 [12]
	AFB1	NaCl<br>MeOH:water 80:20 (v/v)	Aflatest IAC			0.100; 0.300	
	AFM1	NaCl	Aflatest IAC			ND; 0.015	
		NaCl<br>dichloromethane:acetone 1:1 (v/v)					
	PAT	Na2SO4 + NaHCO3 + ethyl acetate/hexane 60:40 (v/v)	Strata ® SPE C18-E		HPLC-UV	ND; 15	

Table S1 (continued)

Matrix	Mycotoxin	Sample pre-treatment		Method			Reference
		Extraction	Clean-up	Derivatization	Detection method	LOD; LOQ (µg/kg)	
Infant formulas and baby food	DON<br>NIV<br>FUS-X<br>DAS<br>15acDON<br>3acDON<br>NEO<br>HT2<br>T2	ACN:water 84:16 (v/v)	-	-	LC-MS/MS	1; 10<br>5.5; 15<br>5.5; 20<br>2; 10<br>2; 10<br>2; 10<br>5.5; 15<br>2; 6<br>3; 8	Juan *et al.*, 2014 [12]
	ZON<br>ENB (ENB1/4)<br>ENA (ENA1)<br>BEA	ACN:MeOH 60:40 (v/v)				1.5; 5<br>5: 10<br>5; 10<br>5; 10<br>5; 10	

Table S1 (continued)

Matrix	Mycotoxin	Sample pre-treatment		Method			Reference
		Extraction	Clean-up	Derivatization	Detection method	LOD; LOQ (µg/kg)	
Cereal baby food (maize, wheat, rice, barley, rye, oat, sorghum, millet, spelt)	VER	QuEChERS	d-SPE	Tri-SilTBT<br>BSA + TMSI + TMCS (3:3:2)	GC-MS	19.19; 63.33	Pereira et al., 2015 [17]
	DON					0.37; 1.24	
	FUS-X					2.79; 9.22	
	DAS					3.07; 10.12	
	3AcDON					17.28; 57.02	
	15AcDON					2.50; 8.25	
	NIV					5.56; 18.36	
	T2-Tetrol					10.48; 34.59	
	NEO					1.28; 4.23	
	T2-Triol					0.90; 2.96	
	HT2					6.40; 21.13	
	T2					6.76; 22.31	

Table S1 (continued)

Matrix	Mycotoxin	Sample pre-treatment		Method			Reference
		Extraction	Clean-up	Derivatization	Detection method	LOD; LOQ (µg/kg)	
Breakfast cereals for children (maize, wheat, rice, and multi-grain	AFB1<br>AFB2<br>AFG1<br>AFG2<br>AFM1<br>OTA	MeOH:water	IAC	Post column bromination	RP-HPLC-FD	0.001 – 0.011; 0.004 – 0.032	Assunção et al., 2015 [8]
	DON<br>NIV<br>T2-Toxin<br>HT2-Toxin	QuEChERS d-SPE		Tri-SilTBT<br>BSA + TMSI + TMCS (3:3:2)	GC-MS	0.4: 1.2<br>5.6; 18.4<br>6.8; 22.3<br>6.4: 21.1	
	FB1<br>FB2	MeOH:water (3:1:100 mL)	SAX SPE cartridge	-	UPLC-MS	0.8; 2.5	

Table S1 (continued)

Matrix	Mycotoxin	Sample pre-treatment		Method			Reference
		Extraction	Clean-up	Derivatization	Detection method	LOD; LOQ (µg/kg)	
Baby food (cereal based supplementary foods for infants and children)	OTA	MeCH:water	-	-	Competitive ELISA (absorbance)	0.025; NM	Hampikyan et al., 2015 [23]
		MeCH:water	IAC OchraTest®	-	HPLC-FD	0.006; 0.019	
Children cereal-based food	PAT	Sodium sulphate + sodium hydrogenocarbonate + ethyl acetate	SPE	-	RP-HPLC-DAD	0.9; 2.9	Assunção et al., 2016 [32]
	OTA	MeOH:water (80:20) + PBS	IAC (AflaOchra, Vicam®)	-	RP-HPLC-FD	0.006; 0.019	

Table S1 (continued)

Matrix	Mycotoxin	Sample pre-treatment		Method			Reference
		Extraction	Clean-up	Derivatization	Detection method	LOD; LOQ (µg/kg)	
Breakfast cereals	AFB1<br>AFB2<br>AFG1<br>AFG2<br>AFM1<br>OTA	MeOH:water (80:20)	IAC (AflaOchra, Vicam®)	-	RP-HPLC-FD	0.003; 0.009<br>0.004; 0.012<br>0.006: 0.018<br>0.010; 0.029<br>0.011; 0.032<br>0.006; 0.019	Martins et al., 2018 [14]
	DON<br>NEO<br>DAS<br>FUS-X<br>15acDON<br>T-2 Triol<br>NIV<br>T-2<br>HT-2<br>3acDON<br>VER<br>T2-Tetrol	QuEChERS d-SPE		BSA + TMCS + TMSI (3:2:3)	GC-MS	0.400; 1.20<br>1.30; 4.2<br>3.1; 10.1<br>2.8; 9.2<br>2.5; 8.3<br>0.9; 3.0<br>5.6; 18.4<br>6.8; 22.3<br>6.4; 21.1<br>17.3; 57.0<br>19.2; 63.3<br>10.5; 34.6	
	FB1<br>FB2<br>ZEA	MeOH:water (75:25)	-	-	UPLC-MS/MS	0.060; 0.180<br>0.120; 0.360<br>0.12; 0.40	

Table S1 (continued)

Matrix	Mycotoxin	Sample pre-treatment		Method			Reference
		Extraction	Clean-up	Derivatization	Detection method	LOD; LOQ (µg/kg)	
Cereal-based children food	AFB1<br>AFB2<br>AFG1<br>AFG2<br>AFM1<br>OTA	MeOH:water (80:20)	IAC (AflaOchra, Vicam®)	-	RP-HPLC-FD	0.003; NM<br>0.001; 0.004<br>0.006; NM<br>0.010; NM<br>0.011; NM<br>0.006; NM	Assunção et al., 2018 [13]
	DON<br>NIV<br>T-2<br>HT-2	QuEChERS d-SPE		BSA + TMCS + TMSI (3:2:3)	GC-MS	0.37; NM<br>5.56; NM<br>6.8; 22.3<br>6.4; NM	
	FB1<br>FB2<br>ZEA	MeOH:water (75:25)	-	-	UPLC-MS/MS	0.08; NM<br>0.08; NM<br>0.12; 0.40	
Cereal-based baby food	AOH<br>AME<br>TEN<br>ATX I<br>ATLP<br>TA	ACN:water (84:16) + formic acid<br>ACN:MeOH:water (50:25:25) + formic acid	SPE	-	UHPLC-MS/MS	0.50; 1.81<br>0.05; 0.23<br>0.05; 0.16<br>0.42; 1.49<br>0.31; 1.03<br>1.25; 4.13	Gotthardt et al., 2019 [9]

Table S1 (continued)

Matrix	Mycotoxin	Sample pre-treatment		Method			Reference
		Extraction	Clean-up	Derivatization	Detection method	LOD; LOQ (µg/kg)	
Cereal-based baby food	AFB1<br>AFB2<br>AFG1<br>AFG2	MeOH:water (80:20)	IAC (AflaTest, Vicam®)	-	HPLC-FD	0.02; 0.06	Herrera et al., 2019 [15]
	DON	MeOH	IAC (DonTest, Vicam®)	-	HPLC-DAD	33; 100	
Cereal-based infant and children food	DON<br>NIV<br>ZEA<br>OTA<br>HT-2<br>T-2<br>FB1<br>FB2	MeCN:acetic acid:water (80:1:19)	-	-	HPLC-MS/MS	2.0; 6.5<br>18.6; 61.9<br>6.1; 20.5<br>0.07; 0.24<br>1.1; 3.7<br>0.1; 0.3<br>1.4; 0.4<br>0.5; 1.5	Postupolski et al., 2019 [29]
Breakfast cereals<br>Sweet snacks	OTA	dichloromethane			ELISA (I'screen Ochra-cod.OR 360)	0.5; 1.0	Capei et al., 2019 [24]

Table S1 (continued)

Matrix	Mycotoxin	Sample pre-treatment		Method			Reference
		Extraction	Clean-up	Derivatization	Detection method	LOD; LOQ (µg/kg)	
Cereal-based infant food	AFL	MeOH:H_2O:acetic acid (79:20:1)	-	-	LC-MS/MS	0.1; 0.25	Braun et al., 2021 [30]
	AFB1					0.15; 0.3	
	STG					0.05; 0.1	
	ZEN					0.3; 0.6	
	DON					10.0; 20.0	
	NIV					8.0; 16.0	
	T-2					0.3; 0.6	
	BEA					0.2; 0.4	
	ENA					0.2; 0.4	
	ENA1					0.2; 0.4	
	ENB					0.2; 0.4	
	ENB1					0.2; 0.4	
	FB1					3.5; 7.0	
	AME					0.3; 0.6	
	TA					12.0; 24.0	
	TTX*					0.5; 1.0	
	ATPL					5.0; 10.0	

AFB1 (Aflatoxin B1), AFB2 (Aflatoxin B2), AFG1 (Aflatoxin G1), AFG2 (Aflatoxin G2), AFM1 (Aflatoxin M1), OTA (Ochratoxin A), DON (Deoxynivalenol), 3acDON (3-acetyldeoxynivelanol); 15acDON (15-acetyldeoxynivelanol), NIV (Nivalenol), FUS-X (Fusarenon-x),T-2 (Mycotoxin T-2), HT-2 (Mycotoxin HT-2), T2-Tetrol (Mycotoxin T2-tetrol), β-ZOL (β-zearalenol), FB1 (Fumonisin B1), FB2 (Fumonisin B2), PAT (Patulin), ZEA (Zearalenone) ENB (Enniatin B), ENB1 (Enniatin B1), ENB2 (Enniatin B2), ENB4 (Enniatin B4), ENA (Enniatin A), ENA1 (Enniatin A1), ENA2 (Enniatin A2), BEA (Beauvericin), STG (Sterigmatocystin), NEO (Neosolaniol), AOH (Alternariol), AME (alternariol monomethyl ether), TEN (Tentoxin), ATX I (Altertoxin 1), ATLP (Alterperylenol), TA (Tenuazonic acid) and AFL (Aflatoxicol); ACN – Acetonitrile; BSA - N,O-bis(trimethylsilyl) acetamide; d-SPE - Dispersive solid-phase extraction; ELISA - Enzyme-linked immunosorbent assay; GC-MS - Gas Chromatography coupled to mass spectrometry; HPLC-FD - High performance liquid chromatography coupled with fluorescence detector; HPLC-UV - High performance liquid chromatography coupled with ultraviolet detector; IAC - Immunoaffinity column; LC-MS/MS - Liquid chromatography coupled to tandem mass spectrometry; MeOH – Methanol; NaCl – Sodium chloride; QuEChERS – Quick, Easy, Cheap, Effective, Rugged and Safe; RP-HPLC-DAD - Reversed-phase high performance liquid chromatography coupled with diode array detector; RP-HPLC-FD - Reversed-phase high performance liquid chromatography coupled with fluorescence detector; SPE - Solid-phase extraction; TMCS – Trimethylchlorosilane; TMSI - N-trimethylsilyimidazole; UHPLC - Ultra high-performance liquid chromatography; UPLC-MS - Ultra performance liquid chromatography coupled with mass spectrometry; UPLC-MS/MS - Ultra performance liquid chromatography coupled with tandem mass spectrometry; NA – not applicable; NM – not mentioned; ND – not detected

References

1. Rahmani, A.; Jinap, S.; Soleimany, F., Qualitative and Quantitative Analysis of Mycotoxins. Compr. Rev. Food Sci. Food Saf 2009, 8 (3), 202-251.

2. Alshannaq, A.; Yu, J. H., Occurrence, Toxicity, and Analysis of Major Mycotoxins in Food. Int J Environ Res Public Health 2017, 14 (6).

3. Shanakhat, H.; Sorrentino, A.; Raiola, A.; Romano, A.; Masi, P.; Cavella, S., Current methods for mycotoxins analysis and innovative strategies for their reduction in cereals: an overview. J. Sci. of Food Agric. 2018, 98 (11), 4003-4013.

4. Xie, L.; Chen, M.; Ying, Y., Development of Methods for Determination of Aflatoxins. Crit. Rev. Food Sci Nutr 2016, 56 (16), 2642-2664.

5. Turner, N. W.; Bramhmbhatt, H.; Szabo-Vezse, M.; Poma, A.; Coker, R.; Piletsky, S. A., Analytical methods for determination of mycotoxins: An update (2009–2014). Anal. Chim. Acta 2015, 901, 12-33.

6. Bueno, D.; Istamboulie, G.; Muñoz, R.; Marty, J., Determination of Mycotoxins in Food: A Review of Bioanalytical to Analytical Methods. Appl. Spectrosc. Rev. 2015, 50 (9), 728-774.

7. Turner, N. W.; Subrahmanyam, S.; Piletsky, S. A., Analytical methods for determination of mycotoxins: a review. Anal. Chim. Acta 2009, 632 (2), 168-80.

8. Assunção, R.; Vasco, E.; Nunes, B.; Loureiro, S.; Martins, C.; Alvito, P., Single-compound and cumulative risk assessment of mycotoxins present in breakfast cereals consumed by children from Lisbon region, Portugal. Food Chem. Toxicol. 2015, 86, 274-281.

9. Gotthardt, M.; Asam, S.; Gunkel, K.; Moghaddam, A. F.; Baumann, E.; Kietz, R.; Rychlik, M., Quantitation of Six Alternaria Toxins in Infant Foods Applying Stable Isotope Labeled Standards. Front. Microbiol. 2019, 10, 109-109.

10. Pereira, V. L.; Fernandes, J. O.; Cunha, S. C., Mycotoxins in cereals and related foodstuffs: A review on occurrence and recent methods of analysis. Trends Food Sci Technol. 2014, 36 (2), 96-136.

11. Krska, R.; Schubert-Ullrich, P.; Molinelli, A.; Sulyok, M.; MacDonald, S.; Crews, C., Mycotoxin analysis: An update. Food Addit. Contam. Part A 2008, 25 (2), 152-163.

12. Juan, C.; Raiola, A.; Mañes, J.; Ritieni, A., Presence of mycotoxin in commercial infant formulas and baby foods from Italian market. Food Control 2014, 39, 227-236.

13. Assunção, R.; Martins, C.; Vasco, E.; Jager, A.; Oliveira, C.; Cunha, S. C.; Fernandes, José O.; Nunes, B.; Loureiro, S.; Alvito, P., Portuguese children dietary exposure to multiple mycotoxins – An overview of risk assessment under MYCOMIX project. Food Chem. Toxicol. 2018, 118, 399-408.

14. Martins, C.; Assunção, R.; Cunha, S. C.; Fernandes, J. O.; Jager, A.; Petta, T.; Oliveira, C. A.; Alvito, P., Assessment of multiple mycotoxins in breakfast cereals available in the Portuguese market. Food Chem. 2018, 239, 132-140.

15. Herrera, M.; Bervis, N.; Carraminana, J. J.; Juan, T.; Herrera, A.; Arino, A.; Loran, S., Occurrence and Exposure Assessment of Aflatoxins and Deoxynivalenol in Cereal-Based Baby Foods for Infants. Toxins (Basel) 2019, 11 (3).

16. Wilson, T. J.; Romer, T. R., Use of the Mycosep Multifunctional Cleanup Column for Liquid Chromatographic Determination of Aflatoxins in Agricultural Products. J. Assoc. Off. Anal. Chem. 1991, 74 (6), 951-956.

17. Pereira, V. L.; Fernandes, J. O.; Cunha, S. C., Comparative assessment of three cleanup procedures after QuEChERS extraction for determination of trichothecenes (type A and type B) in processed cereal-based baby foods by GC–MS. Food Chem. 2015, 182, 143-149.

18. Cunha, S. C.; Fernandes, J. O., Development and validation of a method based on a QuEChERS procedure and heart-cutting GC-MS for determination of five mycotoxins in cereal products. J. Sep. Sci. 2010, 33 (4-5), 600-609.

19. Cunha, S. C.; Fernandes, J. O., Chapter 21 - Application in Food Analysis. In Liquid-Phase Extraction, Poole, C. F., Ed. Elsevier, Amsterdam, Netherlands: 2020; pp 643-665.

20. Tamura, M.; Uyama, A.; Mochizuki, N., Development of a Multi-mycotoxin Analysis in Beer-based Drinks by a Modified QuEChERS Method and Ultra-High-Performance Liquid Chromatography Coupled with Tandem Mass Spectrometry. Anal. Sci. 2011, 27 (6), 629-629.

21. Zheng, M. Z.; Richard, J. L.; Binder, J., A Review of Rapid Methods for the Analysis of Mycotoxins. Mycopath. 2006, 161 (5), 261-273.

22. Shephard, G. S., Current Status of Mycotoxin Analysis: A Critical Review. J. AOAC Intern. 2016, 99 (4), 842-848.

23. Hampikyan, H.; Bingol, E. B.; Colak, H.; Cetin, O.; Bingol, B., Determination Of Ochratoxin A In Baby Foods By ELISA And HPLC. Acta Alimentaria 2015, 44 (4), 578-584.

24. Capei, R.; Pettini, L.; Mando Tacconi, F., Occurrence of Ochratoxin A in breakfast cereals and sweet snacks in Italy: dietary exposure assessment. Ann Ig 2019, 31 (2), 130-139.

25. Boyer, R. F., Modern Expeimental Biochemestry, 3rd Edition. Edited by Cummings B, San Francisco CA. 2000.

26. Bessaire, T.; Mujahid, C.; Mottier, P.; Desmarchelier, A., Multiple Mycotoxins Determination in Food by LC-MS/MS: An International Collaborative Study. Toxins (Basel) 2019, 11 (11).

27. Korfmacher, W. A., Foundation review: Principles and applications of LC-MS in new drug discovery. Drug Discov. Today 2005, 10 (20), 1357-1367.

28. Wang, X.; Wang, S.; Cai, Z., The latest developments and applications of mass spectrometry in food-safety and quality analysis. TrAC Trends Anal. Chem. 2013, 52, 170-185.

29. Postupolski, J.; Starski, A.; Ledzion, E.; Kurpinska-Jaworska, J.; Szczesna, M., Exposure assessment of infants and young children on selected Fusarium toxins. Rocz Panstw Zakl Hig 2019, 70 (1), 5-14.

30. Braun, D.; Eiser, M.; Puntscher, H.; Marko, D.; Warth, B., Natural contaminants in infant food: The case of regulated and emerging mycotoxins. Food Control 2021, 123, 107676.

31. Santos Pereira, C.; C Cunha, S.; Fernandes, J. O., Prevalent Mycotoxins in Animal Feed: Occurrence and Analytical Methods. Toxins 2019, 11 (5), 290.

32. Assunção, R.; Martins, C.; Dupont, D.; Alvito, P., Patulin and ochratoxin A co-occurrence and their bioaccessibility in processed cereal-based foods: A contribution for Portuguese children risk assessment. Food Chem. Toxicol. 2016, 96, 205-214.
